# Ablative Treatments for Small Renal Masses and Management of Recurrences: A Comprehensive Review

**DOI:** 10.3390/life14040450

**Published:** 2024-03-28

**Authors:** Achille Aveta, Vincenzo Iossa, Gianluca Spena, Paolo Conforti, Giovanni Pagano, Fabrizio Dinacci, Paolo Verze, Celeste Manfredi, Matteo Ferro, Francesco Lasorsa, Lorenzo Spirito, Luigi Napolitano, Antonio Tufano, Alessandra Fiorenza, Pierluigi Russo, Fabio Crocerossa, Giuseppe Lucarelli, Sisto Perdonà, Roberto Sanseverino, Salvatore Siracusano, Simone Cilio, Savio Domenico Pandolfo

**Affiliations:** 1 Department of Neurosciences and Reproductive Sciences and Odontostomatology, University of Naples “Federico II”, 80131 Naples, Italy; achille-aveta@hotmail.it (A.A.); pconforti8@gmail.com (P.C.); giovanni.pagano1@outlook.it (G.P.); fabrizio.dinacci18@gmail.com (F.D.); luiginap89@gmail.com (L.N.); alessandra_fiorenza@hotmail.it (A.F.); simocilio.av@gmail.com (S.C.); 2 Department of Urology, Umberto I Hospital, ASL Salerno, 84014 Nocera Inferiore, Italy; vincenzoiossa@msn.com (V.I.); roberto.sanseverino@libero.it (R.S.); 3 Department of Urology, University of L’Aquila, 67100 L’Aquila, Italy; salvatore.siracusano@univaq.it; 4 Department of Urology, Istituto Nazionale Tumori, IRCCS, “Fondazione G. Pascale”, 80131 Naples, Italy; spena.dr@gmail.com (G.S.); antonio.tufano91@gmail.com (A.T.); s.perdona@istitutotumori.na.it (S.P.); 5 Department of Medicine and Surgery, Scuola Medica Salernitana, University of Salerno, 84081 Fisciano, Italy; pverze@gmail.com; 6 Unit of Urology, Department of Woman, Child and General and Specialized Surgery, University of Campania “Luigi Vanvitelli”, 80131 Naples, Italy; manfredi.celeste@gmail.com (C.M.); lorenzospirito@msn.com (L.S.); 7 Division of Urology, European Institute of Oncology, IRCCS, 71013 Milan, Italy; drmatteoferro@gmail.com; 8 Department of Precision and Regenerative Medicine and Ionian Area-Urology, Andrology and Kidney Transplantation Unit, University of Bari “Aldo Moro”, 70124 Bari, Italy; francesco-lasorsa96@libero.it (F.L.); giuseppe.lucarelli@inwind.it (G.L.); 9 Department of Urology, Fondazione Policlinico Universitario Agostino Gemelli, Largo Francesco Vito 1, 00168 Rome, Italy; pierluigi.russo01@icatt.it; 10 Division of Urology, Magna Graecia University of Catanzaro, 88100 Catanzaro, Italy; fabiocrocerossa@yahoo.it

**Keywords:** RCC, small renal masses, ablative techniques, recurrence

## Abstract

This review focuses on ablative techniques for small renal masses (SRMs), including radiofrequency ablation (RFA), cryoablation (CA), microwave ablation (MWA), and irreversible electroporation (IRE), and discusses recurrence management. Through an extensive literature review, we outline the procedures, outcomes, and follow-up strategies associated with each ablative method. The review provides a detailed examination of these techniques—RFA, CA, MWA, and IRE—elucidating their respective outcomes. Recurrence rates vary among them, with RFA and CA showing comparable rates, MWA demonstrating favorable short-term results, and IRE exhibiting promise in experimental stages. For managing recurrences, various strategies are considered, including active surveillance, re-ablation, or salvage surgery. Surveillance is preferred post-RFA and post-CA, due to slow SRM growth, while re-ablation, particularly with RFA and CA, is deemed feasible without additional complications. Salvage surgery emerges as a viable option for larger or resistant tumors. While ablative techniques offer short-term results comparable to surgery, further research is essential to understand their long-term effects fully. Decisions concerning recurrence management should consider individual and tumor-specific factors. Imaging, notably contrast-enhanced ultrasounds, plays a pivotal role in assessing treatment success, emphasizing the necessity of a multidisciplinary approach for optimal outcomes. The lack of randomized trials highlights the need for further research.

## 1. Introduction

Renal cell carcinoma (RCC) is the 14th most prevalent malignancy worldwide, according to the Global Cancer Observatory [1]. Recently, developments in radiology have markedly increased the incidence of RCC. In particular, the spread of ultrasounds (US), computed tomography (CT) scans, and magnetic resonance imaging (MRI) increased detection rates of incidentalomas and small renal masses (SRMs), defined by dimensions inferior to 4 cm [2,3,4,5].

**Figure 1:**
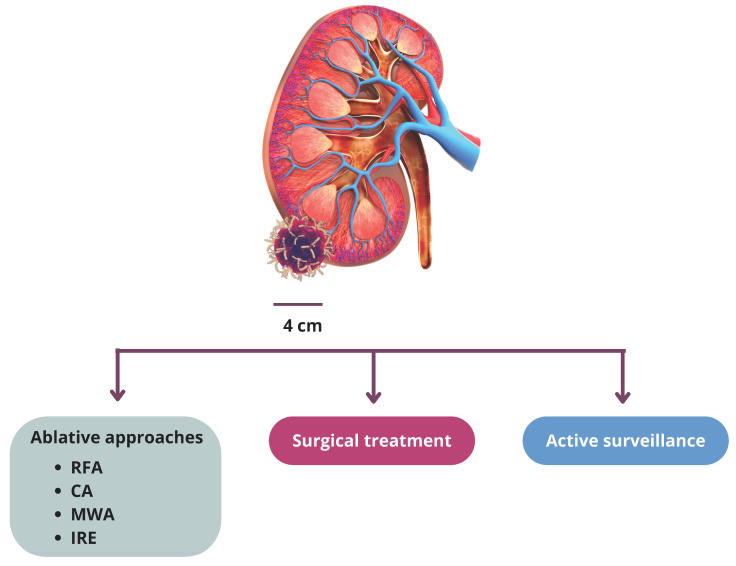
Treatment options for SRMs. SRMs, small renal masses; RFA, radiofrequency ablation; CA, cryoablation; MWA, microwave ablation; IRE, irreversible electroporation.

Various strategies are applicable for managing clinically localized SRMs with suspected RCC, including active surveillance, ablation therapy, and surgery (Figure 1) [6]. Current guidelines endorse nephron-sparing surgery (NSS), such as partial nephrectomy (PN), for managing SRMs when technically feasible [2,7]. However, it is widely recognized that SRMs usually are low-grade, slow-progressing tumors with limited metastatic potential [8,9]. To mitigate surgery-related morbidity and preserve renal function, ablative techniques have been developed as an alternative to PN in patients with SRMs [9,10,11,12,13,14,15,16,17]. Particularly, these techniques should be preferred in elderly patients with multiple comorbidities and pre-existing renal conditions, such as chronic kidney disease, hereditary and/or multiple tumors, and cases of solitary kidneys [18,19]. Ablative techniques can utilize either low-temperature methods, such as cryoablation, or high-temperature approaches, like radiofrequency ablation (RFA) and microwave ablation (MWA), along with a nonthermal modality known as irreversible electroporation (IRE) [20,21].

Since 2017, both the American Urological Association (AUA) and the European Association of Urology (EAU) guidelines have recommended ablative techniques as a valid alternative to surgery for pT1a renal masses (<4 cm), except for those located at the hilum or in proximity to the proximal ureter [2,22]. However, despite these recommendations, current literature still lacks randomized clinical trials comparing ablative techniques to NSS; indeed, only observational studies have been conducted for this purpose [9,23]. Nonetheless, on the one hand, previous studies suggest that the outcomes of ablative techniques are comparable to those of surgical resection, with a 5-year cancer-specific survival rate of 95%; on the other hand, it is crucial to note that the risk of local recurrence and metastasis tends to be more prevalent after focal ablations than after surgical approaches [24,25,26,27].

Consequently, the present manuscript aims to provide a narrative review of the scientific literature on (i) current methods adopted as ablative techniques for SRMs; (ii) short- and long-period outcomes after ablative management; (iii) imaging methods for detecting recurrences; and (iv) lastly, focalizing evidence regarding the management of these recurrences.

## 2. Materials and Methods

A comprehensive and narrative literature review was conducted by searching through databases such as PubMed, Embase, and the Cochrane Library, which encompassed publications up to February 2024.

### 2.1. Search Strategy and Data Extraction

The search was structured around key terms pertinent to the objectives of the review. The terms included “small renal masses”, “ablation therapy”, “recurrence”, “radiofrequency ablation”, “cryoablation”, “microwave ablation”, and “irreversible electroporation”. These keywords were selected to ensure a broad and comprehensive retrieval of relevant literatures that cover various aspects of ablative treatments for SRMs, including procedure details, outcomes, and follow-up strategies. 

Our review process aimed to include studies that provide detailed accounts of the procedures, outcomes, and follow-up strategies associated with each ablative technique. To ensure a thorough and unbiased selection, additional articles were identified through manual searches of reference lists from pertinent studies and review articles.

Data extraction was meticulously performed by two authors independently (AA and VI)), with a predefined strategy to resolve any discrepancies that arose. In instances of disagreement, a third author was consulted to reach a consensus (SDP). This collaborative approach ensured the accuracy and reliability of the data included in our review.

### 2.2. Data Synthesis

The gathered data were synthesized to construct a comprehensive overview of the current landscape of ablative techniques for the management of SRMs and the strategies employed in managing recurrences. This synthesis aims to present a narrative that encompasses the breadth of research in the field, highlighting procedural details, comparative outcomes, and advancements in follow-up strategies (Table 1).

## 3. Ablative Techniques

Ablation procedures can be conducted using either laparoscopic or percutaneous approaches, with the latter being preferred due to its shorter operative time and the ability to perform under local anesthesia [28]. Various ablative techniques are available, including those employing low temperature (cryoablation), high temperature (RFA and MWA), or nonthermal modalities, such as (IRE) (Table 2) [29,30]. Hereafter, we provide a brief panoramic of each technique and highlight strengths, limitations, and differences between them.

### 3.1. Radiofrequency Ablation

RFA was the first ablation technique adopted for the treatment of RCC, as reported by Zlotta et al. in the year 1997 [31]. The procedure involves the placement of one or more radiofrequency electrodes into tumoral tissue as guided by US, CT scans, or MRI [32]. These electrodes induce ionic agitation by delivering electrical current, elevating the temperature above 60 °C, ultimately resulting in cell death through coagulative necrosis. The standard RFA protocol involves an initial electrical power of 30–40 W, increased at a rate of 10 W/min, with two breaks/roll-offs during ablation [33]. Studies have shown that RFA provides an excellent local control rate for T1a RCCs, ranging from 91 to 100%. The 5-year overall survival and cancer-specific survival rates among patients with T1 RCC are 97% and 96% to 97%, respectively.

Limitations of RFA are associated with the “heat-sink effect”, wherein heat absorbed by flowing blood or air is carried away from the ablation area, leading to the dissipation of hyperthermia and a reduction in RFA efficacy [32]. Therefore, RFA is considered less effective for tumors larger than 3 cm, centrally located masses, and masses located in proximity to the ureteropelvic junction or large blood vessels [34].

### 3.2. Cryoablation

Cryoablation (CA) is a technique using argon-based cryoprobes to lower the tumor temperature below −40 °C, which will be subsequently thawed by using helium. The procedure involves double freeze–thaw cycles, comprising 15 min of freezing followed by 10 min of thawing [35]. Monitoring the cryoablation process involves real-time imaging of the created “ice ball” through US, CT, or MRI. The temperature on the tumor margin is actively controlled by real-time sensors [36].

As the RFA approach, renal mass CA can be carried out either percutaneously or laparoscopically. However, the current recommendation from the NCCN favors the percutaneous approach, due to its quicker execution and the avoidance of general anesthesia [37]. The laparoscopic approach may be warranted in specific instances, such as with periureteral or upper pole masses that cannot be effectively targeted percutaneously. Compared to RFA, this approach ensures better outcomes for lesions > 3 cm, and, due to the anesthetic properties of the cold, it is less painful for the patient [38]. However, CA is associated with higher risks of bleeding, since blood arteries surrounding the mass cannot be cauterized as in RFA; moreover, the complete freezing of the renal mass, with the creation of an adequate “ice-ball”, requires the insertion of multiple probes, increasing both procedure timing and risks of damaging surrounding tissues [39].

### 3.3. Microwave Ablation

MWA has been approved in the United States since 2008 [40].

Similar to RFA, MWA utilizes electromagnetic waves to generate heat and induce cell death through hyperthermic injury. A needle-like probe (antenna) is intratumorally placed, producing microwave energy that generates an electromagnetic field, resulting in frictional heating exceeding 100 °C [41]. Unlike RFA, MWA excels in heating larger tumor volumes because during RFA, the active heating zone is limited to a few millimeters around the electrode, whereas MWA can heat tissues up to 2 cm away from the antenna [42,43]. MWA also allows the use of multiple antennas to enhance the ablative effect, facilitating the simultaneous ablation of larger or multifocal tumors; this synergistic capability is not available with RFA [42]. However, MWA systems are bulkier than RFA systems and use larger cables, and the antenna may require cooling mechanisms because of potential overheating [44].

Lastly, Klapperich et al. highlighted that MWA, when compared to RFA and CA, may result in increased pain for the patient. However, the results of their study conducted on 96 patients undergoing MWA demonstrated that this technique had minimal impact on renal function and few ablation-related complications [45].

### 3.4. Irreversible Electroporation

IRE represents a novel nonthermal ablation technique that relies on electrical pulses transmitted between electrodes strategically placed in the tumor area, either through a percutaneous approach (under imaging guidance) or during open surgery [46]. The electric field generated induces changes in the electrochemical potential across the cell membrane, destabilizing the lipid bilayer and creating openings termed “nanopores” [47]. This modification alters the permeability of the cellular matrix, leading to cell death. The primary advantage of IRE over other thermal modalities lies in its avoidance of high-temperature-based mechanisms, thereby reducing the risk of collateral damage due to the heat-sink effect near vasculature. Consequently, IRE is considered the safest technique for tumors located in proximity to large vessels [48]. However, clinical experience with IRE is currently limited, contributing to its classification as an experimental method.

## 4. Imaging in Post-Ablative Techniques Follow-Up: Strategies and Timing

The most important point to evaluate after the use of an ablation technique is the completeness of lesion ablation to achieve an effective therapeutic response. Radiologists play a crucial role in distinguishing between complete ablation and local tumor progression (LTP) [49]. 

Currently, there is no consensus on the radiological technique and timing to be used in the follow-up after ablative techniques. Conventional techniques such as CT or MRI are frequently employed to evaluate the therapeutic efficacy of ablation [50]. However, the nephrotoxicity of contrast in CT and the high frequencies in poorly collaborating patients in MRI pose limitations for these procedures [51,52]. Furthermore, although exceedingly rare, the potential occurrence of nephrogenic systemic fibrosis associated with the use of gadolinium-based contrast agents has been observed in patients with chronic renal failure undergoing MRI [53].

In recent years, contrast-enhanced ultrasound (CEUS) emerged as a safe and well-tolerated imaging method with real-time multiplanar imaging using a non-toxic contrast agent [54]. It is a reproducible technique with high predictive values and specificity in evaluating the ablation effect. Compared to CT and MRI, CEUS may offer potential superiority in detecting LTP by providing real-time visualization of the ablation zone, surrounding renal parenchyma, and renal vessels, despite being negatively influenced by intestinal gas and operator dependency, as in conventional ultrasounds [55].

Regardless of the type of instrumental investigation used, various imaging features enable radiologists to interpret follow-up images. Typically, most studies on post-ablation imaging features focus on tumor-enhancement characteristics and size measurement on follow-up CTs or magnetic resonance (MR) images [56,57].

Focal or curvilinear enhancement within or around the tumor often indicates LTP. However, while clear-cell RCCs demonstrate strong enhancement, non-clear-cell RCCs exhibit limited enhancement in case of LTP [58]. 

Distinguishing local recurrence from post-ablation inflammation is particularly crucial, especially in the case of endophytic tumors; it can be challenging to ascertain whether they are completely ablated or not. In this context, it is important to highlight that a persistent contrast enhancement can be detected up to 9 months after the cryoablation, although it may not necessarily indicate malignancy [59]. Indeed, a weak or moderate enhancement surrounding the residual ablated tumor, resembling a geographic lesion, imperatively indicates the need for a percutaneous biopsy to differentiate LTP from post-ablation inflammation. If this result is negative, the possibility of a false negative is considered, and further evaluation with a short-term follow-up is warranted [60,61]. When focal intra-lesion enhancement is present, LTP should be considered, even if the tumor is not growing. In such cases, a percutaneous biopsy targeting the affected area should be contemplated [49].

Furthermore, changes in the dimensions of an SRM during thermal ablation also contribute to predicting the likelihood of LTP [13,62]. As the RCC shrinks, there is a reduction in the probability of local recurrence, owing to the decreased number of viable tumor cells. This phenomenon is more pronounced in cases of RFA and MWA, compared to cryoablation [63].

Establishing the optimal timing for imaging to assess therapeutic efficacy following ablation techniques constitutes another pivotal point in enhancing diagnostic capabilities and improving the tumor-free survival rate. Currently, there is no consensus on the surveillance intervals for repeating imaging; the choice should be based on the characteristics of the lesion and the patient, adjusting it according to the suspicion of LTP [64]. Some authors suggest an initial radiological assessment after 6 weeks, while others delay the first follow-up to 3 months [65,66]. Hoeffel et al. advocate for obtaining the initial imaging evaluation within 24 h from treatment to exclude early complications and for obtaining the second assessment after 6 weeks [67]. Following these early evaluations, most authors agree on a follow-up at 6 or 9 months after the procedure. Moreover, AUA guidelines recommend an assessment every 12 months for approximately 5 years [22].

## 5. Recurrence Rates Following Ablative Techniques

According to the treatment employed, local recurrence (LR) rates range from 1% to 9% [68]. Specifically, LR rates after the aforementioned ablative techniques are higher than those after surgery (2–10% vs. 1–2%) [69]. However, even though PN is superior to ablation in terms of overall survival (OS) and LR, ablative techniques result in lower complication rates [70]. In general, regardless of the technique used (PN vs. Ablation), most recurrences occur within 5 years and rarely decades after primary treatment [71]. Furthermore, LR mostly occurs at the site of the primary treatment within the kidney, while extrarenal local recurrences are uncommon [72].

Regarding CA, Zargar et al. analyzed data from 139 patients undergoing CA with a median follow-up of 24 months. The authors observed LTP in 10 (7.2%) patients and reported that for each increase of 1 cm in tumor size, patients were 1.5 times more likely to have a tumor recurrence [73]. Similarly, Breen et al. analyzed outcomes from ablative CA performed on 171 tumors in 147 patients, reporting an initial incomplete treatment rate of 7.6%, which improved to 2.4% with CA retreatment [74]. Overall, RFA and CA present comparable recurrence rates, which are higher than those associated with PN, yet they exhibit fewer complications than PN, probably due to their minimally invasive nature (RR: 0.72, 95%CI 0.55–0.94, *p* = 0.004) [44]. Typical complications following ablation comprise procedural bleeding, perirenal hematoma, temporary hematuria, and visceral injury. Among these, bleeding stands out as the most frequent complication [75].

In the specific case of RFA, Psutka et al. conducted a retrospective analysis of long-term oncologic outcomes of 185 patients with T1 RCC with an average tumor size of 3 cm. Of them, 143 (77%) presented with T1a tumors and 42 (23%) with T1b tumors. Overall, 12 (6.5%) experienced LR, with 6 out of 143 (4.2%) in T1a patients and 6 out of 42 (14.3%) in T1b patients. Therefore, tumor stage is a significant predictor of the higher risk of recurrence (stage T1b vs. T1a: univariate HR 3.38, *p* = 0.0072; multivariate HR 4.3, *p* = 0.0085) [34]. Similar findings were reported by Lam CJ et al. in a retrospective-fashioned study conducted between October 2011 and May 2019 involving 141 patients. The mean ± standard deviation (SD) tumor size was 2.6 ± 0.8 cm, and the mean follow-up was 67 (81–161) months. After RFA, the authors reported recurrence rates of 6.4% [76].

Similar LR rates have been reported for MWA. Indeed, McClure et al. conducted a meta-analysis to compare outcomes in terms of LR between MWA and more traditional ablative techniques, such as CA. The authors found low rates of LR for MWA, compared to traditional ablative techniques; in particular, these rates ranged from 2% to 5% at 1 year and 5 years, respectively, for MWA, while rates stood at 6% at both times for CA. Consequently, the one-year local recurrence was significantly improved with MWA compared to CA, while at five years, the rates of local recurrence were similar. This short-term lower recurrence rate is probably attributed to the higher intertumoral temperature and larger ablation zone achieved with MWA. Regarding other outcomes, such as overall survival, disease-free survival, overall/major complications, procedure/ablation time, 1- to 3-month primary technique efficacy, and technical success, the authors reported no significant differences between MWA and other techniques [77].

The most recently developed ablation technique is the IRE, which is still considered an experimental technique; indeed, the majority of available studies involve 10 or fewer patients. Additionally, the limited available follow-up times pose a challenge in establishing reliable oncological outcomes, as most studies had a follow-up duration of less than a year, which we consider the minimum necessary duration. The largest study reported in the current literature is by Canvasser et al., in which 41 patients, with a median tumor size of 2 cm, underwent IRE. With a mean follow-up of 22 months, the 2-year local recurrence-free survival (LRFS) was 83%. Therefore, although IRE has low morbidity, according to the available preliminary studies, this technique has suboptimal short-term local disease control when compared with conventional thermal ablation techniques [78]. A Larger series and longer follow-up studies will need to be conducted to determine long-term outcomes [79].

## 6. How to Address SRMs Following Unsuccessful Ablative Therapy?

When SRMs are selected for ablative techniques, the rates of persistence or recurrence are very low. Consequently, there is limited information regarding management strategies on how to address residual tumors following unsuccessful ablative therapy. As discussed by Breda et al. in their review, surgeons have three options at their disposal: active surveillance, repeated ablation, or salvage surgery, typically involving salvage nephrectomy [26].

As mentioned earlier, contrast enhancement after renal ablation does not exclusively indicate a recurrence; on the contrary, it may be an expression of inflammation or volume-averaging discrepancies in imaging [80]. Active surveillance, especially in the case of RFA and CA, appears to be the most appropriate option, considering that even untreated SRMs show growth rates of 0.2 cm per year [81]. Moreover, in case of recurrence after one year, it does not seem to limit or alter future treatment options [82].

Approximately 66% to 73% of patients undergoing initial ablative treatment and experiencing LTR are estimated to undergo repeated ablation [81]. Indeed, approximately 7.4% to 8.5% of all RFA-treated lesions and 0.9% to 1.3% of CA-treated lesions are managed by repeating ablations [83]. The higher rates of re-ablation after RFA may be explained by the type of procedure used for the initial treatment, typically employing a percutaneous approach compared to CA, which is primarily performed using a laparoscopic technique [84]. Firstly, the laparoscopic technique allows for the better placement of the probe under direct vision; secondly, the percutaneous approach presents lower morbidity and risk than repeated laparoscopic procedures. Therefore, physicians are more inclined to repeat the percutaneous procedure than the laparoscopic one [83].

Contrary to expectations, performing a repeated ablation poses no additional technical challenges when compared to the primary ablation procedure [85]. In this regard, Okhunov et al. have reported outstanding outcomes in salvage CA for T1a SMRs, emphasizing that salvage CA is simpler than the primary treatment, due to the presence of identifiable landmarks resulting from the post-ablation tissue reaction, which aids in targeting the tumor more effectively. In their smaller multicentered report, they also demonstrated a 100% cancer-specific survival rate [86]. Similarly, Matin et al. reported a notably low incidence of therapeutic shortcomings, standing at 4.2% among individuals subjected to salvage ablative interventions. Within the subgroup manifesting recurrent pathology, the research revealed an overarching survival rate of 82.5% and a metastasis-free survival rate of 97.4% over a span of two years for patients harboring localized, unilateral tumors [87].

It is crucial to communicate to the patient that ablative salvage procedures generally have the potential for lower success rates. In this context, Loloi et al. documented failure rates of approximately 6% for primary ablation, around 25% for secondary ablation, and approximately 50% for tertiary ablation [88]. Hence, considering on the one hand the rarity of LR and on the other hand the absence of level I recommendations from international guidelines, the decision on the most appropriate approach is still challenging for both clinicians and patients. Generally, surgery is the most suitable therapeutic option in case of large tumor size or disease progression after an initial failed ablative treatment. The choice between PN and radical nephrectomy (RN) depends not only on the type of lesion but also on the patient and, most importantly, on the surgeon’s experience. Accordingly, the fibrosis surrounding the residual tumor resulting from the ablative procedure undoubtedly influences the choice of salvage surgery [89]. This was highlighted in a study provided by Nguyen et al., where in half of the patients initially considered for PN, the procedure was converted to RN due to extensive scarring and fibrosis [90].

In contrast, Kowalczyk et al. stated that open PN could be considered a safe approach in these patients. Indeed, none of the 16 patients operated on were converted; however, PN after radio-frequency ablation had a higher reintervention rate compared to other series of primary or repeated PN [89]. 

In 2010, Breda et al. suggested that PN should be recommended in selected patients after unsuccessful ablative therapy, preferably opting for an open approach. Moreover, in the case of RN, they proposed a laparoscopic approach, considering it equally safe [26].

Additionally, Karam et al. reported oncological outcomes in 14 patients, of whom 11 underwent PN and 3 underwent RN. Most surgeries were performed using an open approach, with one laparoscopic and one robotic-assisted surgery. Over a median follow-up of 26.5 months, no deaths were observed. However, a high rate of overall perioperative complications was recorded, in particular an intra-operative complication in one patient (a pleurotomy) and postoperative complications in nine patients (64%), with four complications being Clavien grade III [91].

Similar rates of complications were observed by Jimenez et al. in 27 patients who underwent salvage surgery. Six patients (22%) experienced major complications (Clavien grade III-IVb), including four with hemorrhagic complications, while minor complications (Clavien grade II) were reported in four patients (15%). They emphasized that salvage surgery is complex but feasible, with adequate preservation of renal function, even in patients with a solitary kidney, severe chronic kidney disease (CKD), or high-complexity tumors [92].

Additional investigations are necessary to assess the long-term effectiveness of salvage techniques, especially concerning robotic surgery. The largest caseload of salvage robotic renal surgery after failed tumor ablation was conducted by Martini et al. They assessed the role of salvage robotic surgery, based on a multi-institutional collaborative dataset promoted by the Junior ERUS/YAU robot-assisted surgery working group of the European Association of Urology. They recorded an intraoperative complication rate of 6% and a postoperative complication rate of 20%. Furthermore, it was revealed that the prior ablative technique did not negatively impact the pathologist’s ability to detect tumor cells in the samples [93]. In conclusion, robotic-assisted surgery proves to be a valuable tool in salvage surgery after ablative treatment when performed by experienced surgeons in high-volume centers [94].

## 7. Limitations

This review has several limitations that warrant attention. Firstly, the non-systematic nature of data collection and analysis limits the ability to perform direct comparisons among the various ablative techniques discussed. Secondly, variations in the methodologies of included studies, including study designs, sample sizes, and follow-up durations, may compromise the uniformity of presented results. Thirdly, most studies rely on retrospective data, which can be subject to selection bias. Lastly, the absence of randomized controlled trials further limits the strength of the conclusions that can be drawn regarding the comparative efficacy and safety of ablative techniques in managing SRMs and in the management of recurrences. Therefore, the findings of this review should be interpreted with caution and considered as a starting point for more in-depth and systematic future research on the topic.

## 8. Conclusions

Ablative techniques offer viable alternatives to surgery, with each presenting unique benefits and considerations. While short-term outcomes demonstrate comparable efficacy between ablative techniques and surgical interventions, the long-term implications, especially regarding local recurrence and metastasis, warrant further investigation. The absence of randomized clinical trials comparing ablative methods to surgery underscores the need for further research to support evidence-based decision-making. The management of residual SRMs following ablative treatment necessitates a discerning approach considering individual patient factors, lesion characteristics, and evolving technological advancements. As the field continues to evolve, a personalized and multidisciplinary approach remains crucial in optimizing outcomes and minimizing the impact of recurrent SRMs.

## Figures and Tables

**Table 1:** The search strategy summary.

Items	Specifications
Databases and other sources searched	PubMed, Embase, and Cochrane Library
Search terms used	small renal masses, ablation therapy, recurrence, radiofrequency ablation, cryoablation, microwave ablation, and irreversible electroporation.
Timeframe	1997–2024
Inclusion criteria	Articles written in English and those reporting outcomes of ablative treatments for SRMs.
Selection process	Two authors were responsible for data collection. Any discrepancies were resolved through discussion with a third author.

**Table 2:** Comparisons between currently available ablative techniques.

Ablative Method	Advantages	Disadvantages
CA	Real-time visualization Lesions >3 cm Less painful than RFA	Longer procedural time More bleeding risk
RFA	Shorter procedural time Less bleeding risk	No real-time visualization No lesions >3 cm More painful than CA “Heat-sink effect”
MWA	Shorter procedural time Lesions >3 cm No “heat sink effect” Simultaneous ablation	More painful No real-time visualization Bulkier than RFA Need for a cooling mechanism
IRE	Avoidance of change in temperature No “heat sink effect” Less risk of vessel damage	Limited clinical experience

Abbreviations: CA: cryoablation; RFA: radiofrequency ablation; MWA: microwave ablation; IRE: irreversible electroporation.

## Data Availability

Not applicable.
